# A Possible Link between the Environment and *Cryptococcus gattii* Nasal Colonisation in Koalas (*Phascolarctos cinereus*) in the Liverpool Plains, New South Wales

**DOI:** 10.3390/ijerph19084603

**Published:** 2022-04-11

**Authors:** Alex Kan, Laura J. Schmertmann, Clare McArthur, Valentina S. A. Mella, Mathew S. Crowther, Luisa Miranda, Richard Malik, Wieland Meyer, Mark B. Krockenberger

**Affiliations:** 1 Sydney School of Veterinary Science, Faculty of Science, The University of Sydney, Camperdown, NSW 2050, Australia; laura.schmertmann@gmail.com (L.J.S.); luisa.miranda@sydney.edu.au (L.M.); 2 Westmead Institute for Medical Research, Westmead, NSW 2145, Australia; wieland.meyer@sydney.edu.au; 3 Molecular Mycology Research Laboratory, Centre for Infectious Diseases and Microbiology, Westmead Clinical School, Sydney Medical School, The University of Sydney, Westmead, NSW 2145, Australia; 4 School of Life and Environmental Sciences, Faculty of Science, The University of Sydney, Camperdown, NSW 2006, Australia; clare.mcarthur@sydney.edu.au (C.M.); valentina.mella@sydney.edu.au (V.S.A.M.); mathew.crowther@sydney.edu.au (M.S.C.); 5 Veterinary Pathology Diagnostic Services, Sydney School of Veterinary Science, Faculty of Science, The University of Sydney, Camperdown, NSW 2050, Australia; 6 Centre for Veterinary Education, The University of Sydney, Camperdown, NSW 2006, Australia; richard.malik@sydney.edu.au; 7 Sydney Institute for Infectious Diseases, The University of Sydney, Westmead, NSW 2145, Australia; 8 Research and Education Network, Western Sydney Local Health District, Westmead, NSW 2145, Australia; 9 Curtin Medical School, Curtin University, Bentley, WA 6102, Australia

**Keywords:** *Cryptococcus*, koalas, tree hollows, environmental sampling, environmental load

## Abstract

Cryptococcosis caused by yeasts of the *Cryptococcus gattii* species complex is an increasingly important mycological disease in humans and other mammals. In Australia, cases of *C. gattii*-related cryptococcosis are more prevalent in the koala (*Phascolarctos cinereus*) compared to humans and other animals, likely due to the close association that both *C. gattii* and koalas have with *Eucalyptus* species. This provides a cogent opportunity to investigate the epidemiology of spontaneous *C. gattii* infections in a free-living mammalian host, thereby offering insights into similar infections in humans. This study aimed to establish a link between nasal colonisation by *C. gattii* in free-ranging koalas and the tree hollows of *Eucalyptus* species, the key environmental source of the pathogen. We (i) detected and genotyped *C. gattii* from nine out of 169 free-ranging koalas and representative tree hollows within their home range in the Liverpool Plains, New South Wales, and (ii) examined potential environmental predictors of nasal colonisation in koalas and the presence of *C. gattii* in tree hollows. Phylogenetic analyses based on multi-locus sequence typing (MLST) revealed that the koalas were most likely colonised by the most abundant *C. gattii* genotypes found in the *Eucalyptus* species, or closely related genotypes. Importantly, the likelihood of the presence of *C. gattii* in tree hollows was correlated with increasing hollow size.

## 1. Introduction

Cryptococcosis is a mycotic disease of humans and other mammalian species. It has become increasingly prevalent over the last two decades [1]. The disease is caused by the environmental basidiomycetous yeasts of the *Cryptococcus gattii* and *Cryptococcus neoformans* species complexes. In this paper, we will adopt the “species complex” nomenclature suggested by Kwon-Chung et al. [2]. Each of these two species complexes can be further classified into molecular types, with the *C. gattii* species complex containing six molecular types—VGI, VGII, VGIII, VGIV, and the recently established VGV and VGVI [3]—and the *C. neoformans* species complex containing five molecular types: VNI, VNB, VNII, VNIII, and VNIV [2,4]. Subsequently, the *C. gattii* species complex will be referred to as ‘*C. gattii*’ and the *C. neoformans* species complex will be referred to as ‘*C. neoformans*’ unless the molecular type is denoted.

Cryptococcosis caused by *C. neoformans* predominantly affects immunocompromised patients, and there are an estimated 1 million cases of *C. neoformans*-related cryptococcosis reported globally per year, closely reflecting the distribution and number of people living with untreated HIV/AIDS [5]. On the other hand, the number of *C. gattii*-related cases is substantially lower, accounting for less than 20% of global cases [6]. However, the isolation of *C. gattii* from both immunocompetent and immunocompromised clinical cases with no observed bias [7] suggests a greater risk to public health in regions where HIV/AIDS is less prevalent. Unanticipated outbreaks of cryptococcosis caused by *C. gattii* VGII in the Pacific-Northwest in the 1990s [8,9], and its spread through nearby regions of North America, has emphasised our limited understanding of the epidemiology of *C. gattii* and its potential to become a globally significant pathogen outside of the ‘classical’ geographical predilections of subtropical and tropical regions.

The environmental, pathogenic, and host factors that predispose patients to infection by these opportunistic pathogens remain largely unclear. The inhalation of infective propagules of *C. gattii* is required for the establishment of invasive disease in humans and animals [10]. The range of environmental sources of *C. gattii* is extensive, from soil, varying tree species and related debris [10], to the wheel wells of vehicles [9] and indoor dust from homes [11]. Although literature identifying and evaluating *C. gattii* found in clinical or veterinary cases, or from environmental isolation are many, few studies draw a direct connection between identified case isolates and a possible source of infection. In Australia, *C. gattii* VGI is the most common molecular type isolated from the environment, and is also the most frequent cause of human and veterinary *C. gattii*-related cryptococcosis [12,13]. The only exceptions are in the Northern Territory and Western Australia, where *C. gattii* VGII is the most prevalent cause of cryptococcosis, particularly in indigenous Australian populations, with Aboriginal individuals accounting for 26.7% of cases in Australia [14,15], although they comprise less than 0.5% of the population. It has previously been established that *C. gattii* is strongly associated with a number of *Eucalyptus* species in Australia [12,16,17], and that it can be isolated from a range of tree material, such as flowers and bark [16]. In particular, the tree hollows of these species have been identified as the most common reservoirs of *C. gattii*, likely due to the abundance of organic detritus, and shelter from weather and sunlight [17,18,19,20]. Presumably due to their close association to *Eucalyptus* species, *C. gattii*-related cryptococcosis is more common in koalas (*Phascolarctos cinereus*) than other mammalian species [21,22]. As a result, koalas provide a valuable model to study the epidemiology of *C. gattii* as a spontaneous animal disease model and provide greater insight into the risk posed to humans who may frequently be exposed to *Eucalyptus* material and nearby environments, such as forestry workers, farmers, and indigenous groups.

Nasal colonisation is the suspected precursor to cryptococcosis, and is the most common outcome of a significant interaction with *C. gattii* [23]. It is defined by the isolation of yeasts from the nasal cavity of healthy animals without detectable antigenemia or evidence of tissue invasion [24]. Colonisation likely occurs through the inhalation of infectious propagules (likely basidiospores) from the environment, followed by subsequent establishment in the mucosal layer of the upper respiratory tract [10,23,24]. Progression towards subclinical disease—such as localised planum nasale or nasopharyngeal lesions—may occur following colonization; in some cases, further dissemination from invasive sino-nasal disease to the lungs or the central nervous system can occur, causing severe tissue injury, including pneumonia or meningoencephalitis [10,23]. Previous studies have demonstrated that colonisation and/or subclinical infection in koalas by *C. gattii* VGI and VGII may occur commonly, whereas clinical disease in koalas and companion species, such as dogs and cats, is rare [23,25,26].

It is suspected that the load of *C. gattii* in the environment may play a major role in the colonisation or infection of humans and other mammals [22,24]. In murine models, a steep dose–response relationship exists, where progression to severe cryptococcosis occurs after inoculation with a threshold dose of *C. gattii* VGI or VGIIb [27], suggesting that a higher environmental load may contribute to a greater likelihood of infection or colonisation. Krockenberger et al. demonstrated an association between the high-grade environmental presence of *C. gattii* and the prevalence of nasal colonisation in captive koalas. They documented the nasal colonisation of a previously negative koala when it was moved to a new captive facility with a greater environmental presence of the yeast [22]. The environmental colonisation of introduced hardwood perches, which previously tested negative for *C. gattii*, occurred when they were placed in enclosures with colonized koalas [22].

A similar case occurred in a zoo in Japan where a previously naïve koala later tested positive for *C. gattii* nasal colonisation, which was suspected to have originated from colonised koalas translocated from Australia [26]. More recently, an outbreak of *C. gattii* VGII occurred as the possible result of the introduction of a colonized koala from Western Australia into a group of co-owned wildlife parks in Queensland, in which the movement and exchange of koalas occurs regularly [28]. This suggests that the transmission of the *C. gattii* may occur through the contamination of the environment by infected/colonized individuals, followed by the propagation of the pathogen in the new environment, with colonization/infection then occurring in naïve individuals which are exposed to high environmental loads. In the case of captive populations, this in turn can be further aggravated by a high stocking density of colonized koalas, based on the notion that koalas can amplify the environmental presence in tree hollows through the effects of faeces, urine and scarification by their claws [29]. We predicted, in contradistinction, that the effect of the environmental load in natural environments would be greatly attenuated compared to the captive environments investigated in previous studies due to the much lower animal densities and greater habitat dispersal. Nonetheless, the environmental load of *C. gattii* may still play a significant role in the colonisation of wild koalas.

The examination of the environmental factors that affect the presence and load of *C. gattii* becomes important in the establishment of areas of high risk for infection. Interregional factors, such as climatic variation, have been repeatedly examined as limiters of environmental *C. gattii*. For example, winter temperatures below 0 °C and heavy summer rainfall in the outbreak areas of British Columbia, the Pacific Northwest of the USA, and Europe significantly mitigate the presence of *C. gattii* in the environment [30,31,32]. However, the influence of the climate seems to have little effect on the presence of *C. gattii* in New South Wales (NSW), Australia, and cannot explain the large variation in the presence of *C. gattii* between similar habitats within the same region [22]. This suggests that other variables that predict the presence of *C. gattii* must exist between these habitats.

*C. gattii* has a predisposition to colonise certain tree genera, such as *Eucalyptus* spp. and *Ceratonia* spp. [12,17,31,33], but whether there is a tendency to colonise specific species within a genus has not been thoroughly investigated. Other tree factors, such as age or variables affecting the tree hollows themselves, e.g., exposure to sunlight, have not yet been examined but may explain the large variations in *C. gattii* presence between habitats or even individual trees.

We investigated the prevalence of *C. gattii* within a free-ranging population of koalas and tree hollows of *Eucalyptus* species within their home ranges in the Liverpool Plains region of northern NSW, Australia. The ISHAM consensus multi-locus sequence typing (MLST) scheme [34] was employed to genotype environmental and koala isolates, in order to identify shared genotypes, elucidate the phylogenetic relationship between the two groups, and identify possible colonisation/disease-associated strains. In addition, we employed the systematic sampling of tree hollows for *Eucalyptus* species, along transects overlapping known home ranges of GPS-tracked koalas. By doing so, we aimed to compare the environmental load and presence of *C. gattii* between the home ranges of naïve koalas and koalas with nasal colonisation, and to evaluate the role of different predictors of *Eucalyptus* trees and hollows that may affect the likelihood of hollow colonization by *C. gattii*.

## 2. Materials and Methods

### 2.1. Animal Sampling

A total of 169 free-ranging koalas were captured at two sites approximately 50 km apart, the Dimberoy (31°08′24″ S 150°01′34″ E) and Watermark (31°15′57″ S 150°22′16″ E) sites, located in the Liverpool Plains region of NSW, during half-yearly field trips between 2015 and 2017. The selected koalas were ear-tagged and fitted with a Sirtrack GPS collar (Sirtrack Ltd., Hawkes Bay, New Zealand) weighing less than 3% of their body weight at capture. In order to determine the nasal colonization status of collared koalas, a sterile saline-moistened cotton swab was inserted approximately 2–2.5 cm into both nasal vestibules (left and right) and thoroughly rotated against the inner mucosal surface. The swabs were stored in Amies Transport Medium in the dark at room temperature during the field collection, and were then transferred to refrigerated storage for a maximum of five days prior to plating.

The GPS co-ordinates of the koalas were recorded every 4 h for up to 5 months. Further detailed information on the GPS tracking methods can be found in Crowther et al., 2014 [35] and Rus et al., 2021 [36].

All of the procedures were undertaken with approval from The University of Sydney Animal Ethics Committee (Project number 2016/955) and under veterinarian supervision.

### 2.2. Environmental Sampling

The environmental sampling was conducted during three field trips over four months (February–May 2017) on transects at the two koala capture sites, the Watermark site and the Dimberoy site.

From the data obtained from the animal sampling and GPS tracking, 14 transects (Transects 1–14) of 250–600 m in length (dependent on tree density and home range size) and at least 150 m apart were designed using the geographical information software ArcGIS Ver. 10.5.1 (Esri, Redlands, CA, USA), intersecting trees within known home ranges of koalas with and without nasal colonisation. The start and endpoints of the transects were then uploaded on portable GPS devices (Garmin eTrex 10, Garmin, Lenexa, KS, USA) and used as a guide to sample trees within 5 m of these transects. An additional four transects were conducted opportunistically without koala GPS tracking data (Transects 15–18) but in areas suspected to be within a koala habitat, or where koalas were previously captured and assigned a GPS collar but had not yet been recaptured. GPS data were later obtained for koalas on two of these transects (Transect 15 and Transect 18). In total, 16/18 of the transects were conducted within known koala home ranges. Of these transects, 5/16 represented koalas with colonisation, and the remaining represented koalas without colonization. The opportunistic environmental sampling of trees where koalas were caught was also undertaken (Supplementary Table S1).

Environmental samples were obtained from tree hollows of *Eucalyptus* species in the form of swabs and bagged debris, as described by Krockenberger et al. [22]. Sterile saline-moistened cotton swabs were rotated over the inner surface of the tree hollows, covering as much surface area as possible. The swabs were stored in Amies Transport Medium. The bagged debris was obtained by collecting two generous scoops of organic debris using a hand trowel from tree hollows if it was present, into a clean, sealed, plastic bag. The hand trowel was wiped with 70% ethanol between the collections of the debris. Surface debris was avoided, while deeper organic debris—approximately 2 cm below the surface—was preferred when sampling. Both the swabs and bagged debris were kept in the dark at room temperature for a maximum of three days during collection, and were subsequently moved to 4 °C storage for a maximum of nine days prior to the culturing.

Individual measurements of tree height and tree diameter were recorded. Measurements of the tree hollow itself were taken, including the hollow height from the ground and the directional aspect of the hollow opening, as this would affect the exposure of the hollow to sunlight. Observations of the hollow directional aspect were used to generate a sun index, i.e., a relative representation of sun exposure categorised into five levels: north-facing hollows scored the highest value of 5, northeast- and northwest-facing hollows scored 4, east and west facing hollows scored 3, southeast- and southwest facing hollows -scored 2, and south-facing hollows scored 1. The size of the hollow was hypothesised to affect the overall exposure to weather and the availability of organic detritus. The hollow size was divided into three categories, which were dependent on the diameter of the hollow opening: small (0–15 cm), medium (15 cm–30 cm), and large (>30 cm) (Supplementary Table S1).

### 2.3. Mycological Culture

Each environmental and animal sample was cultured on Staib’s Bird Seed (*Guizotia abyssinica*) Extract Agar (BSA), and was incubated for 7 days at 27 °C. BSA was used to identify *C. gattii*/*C. neoformans* colonies from those of other environmental micro-organisms by the brown-colour-effect [37]. Environmental and animal swabs were directly inoculated onto the BSA by rotating the swab over the surface of the agar. For the environmental bagged debris, a sterile, moistened, cotton swab was introduced into the debris, and was subsequently used to inoculate the BSA.

After incubation on BSA, colony-forming unit (CFU) counts were conducted on plates displaying colonies with the brown-colour-effect, categorised into three categories of colonisation load (<20, 20–100, and >100 CFU/plate), and single colonies were subsequently subcultured onto Sabouraud’s dextrose agar (SDA). At least one and up to twelve individual colonies were isolated on separate SDA agar plates from each positive sample, dependent on the level of colonisation on each plate, and were incubated at 37 °C for 48–72 h prior to the DNA extraction.

### 2.4. DNA Extraction

The DNA extraction was conducted using a modified version of the Ferrer et al. DNA extraction protocol for fungal cultures [38]. For each isolate, a generous amount (1–2 inoculation loops) of fresh colony material (24–72 h) was transferred from the SDA plate to a 1.5 µL Eppendorf tube containing 250 µL sterile distilled water, and was stored at −20 °C for a minimum of 1 h. The samples were then incubated at 65 °C with 500 µL Lysis buffer (0.5 g SDS, 1.4 g NaCl, 0.73 g EDTA, 20 mL Tris-HCL 1 M, 80 mL dH_2_O) and 5 µL 2-mercaptoethanol for 1 h. In total, 500 µL phenol-chloroform-isoamyl alcohol (25:24:1, vol/vol/vol) was then added to the sample and vigorously vortexed to produce a homogenous suspension. The suspension was then centrifuged at 14,000 rpm for 15 min. In total, 500 µL of the upper aqueous layer of the resulting solution was mixed with an equal volume of isopropanol and precipitated for a minimum of 1 h at −30 °C. The sample was then centrifuged at 14,000 rpm at 4 °C for 15 min before the supernatant was discarded. The resulting pellet was then washed with 70% ethanol and centrifuged again at 14,000 rpm for 15 min. The supernatant was then removed, and the pellet was dried before being resuspended in 50 µL sterile distilled water.

### 2.5. Molecular Typing

The PCR amplification of the *URA5* locus was performed for each isolate to be used in the restriction fragment length polymorphism (RFLP) analysis [39]. Each PCR solution contained 10 µL DNA (10 ng/µL) and 40 µL master mix (5 µL 10x Reaction Buffer (Bioline Pty Ltd., London, UK), 5 µL dNTP (2 mmol) (Bioline Pty Ltd., London, UK), 5 µL URA5 Forward Primer (10 ng/µL) (5′ ATGTCCTCCCAAGCCCTCGAC 3′), 5 µL URA5 Reverse Primer (10 ng/µL) (5′ TTAAGACCTCTGAACACCGTACTC 3′), 3 µL MgCl_2_ (50 mmol) (Bioline Pty Ltd., London, UK), 16.5 µL dH_2_O, 0.5 µL BioTaq (Bioline Pty Ltd., London, UK). The target was amplified following the conditions of pre-heating at 94 °C for 3 min, 35 cycles of denaturation at 94 °C for 45 s, annealing at 63 °C for 1 min, and extension at 72 °C for 2 min.

The PCR products from each isolate and the standard reference strains for the eight major molecular types of the *C. gattii*/*C. neoformans* species complexes (WM 148 (VNI), WM 626 (VNII), WM 628 (VNIII), WM 629 (VNIV), WM 179 (VGI), WM 178 (VGII), WM 161 (VGIII), and WM 779 (VGIV)) underwent enzyme digestion by mixing 25.8 µL *URA5* PCR product with 4.2 µL RFLP master mix (3 µL NE buffer 4, 0.3 µL purified BSA 100x, 0.3 µL *HhaI*, 0.6 µL *Sau96I*), followed by incubation at 37 °C for 3 h before being visualised on a 2.5% agarose gel run at 80 V for 3 h.

### 2.6. MLST and Sequencing

The PCRs conducted in this study followed the protocols and conditions provided by the ISHAM consensus MLST scheme for the *C. neoformans*/*C. gattii* species complexes for all seven loci required in the scheme (available at https://mlst.mycologylab.org/ (accessed on 31 August 2017)) [34]. The PCR products for each locus were sent to Macrogen Inc. (Seoul, South Korea) for sequencing. The resulting sequence reads were then processed and trimmed to the correct start (5′) and end (3′) points using the Sequencher ver. 5.4.6 DNA sequence analysis software (Gene Codes Corporation, Ann Arbor, MI, USA). The sequence type (ST)—a composite code of all seven MLST loci allele types (AT) for each sample—was assigned using the Fungal MLST database (Available at https://mlst.mycologylab.org/ (accessed on 31 August 2017)). In the case of new AT combinations, the curators of the MLST database were contacted for the designation of a new ST.

### 2.7. Phylogenetic Analysis

Phylogenetic analysis was conducted using the Molecular Evolutionary Genetics Analysis (MEGA) Ver. 7 software (Pennsylvania State University, State College, PA, USA) on the concatenated sequences of all seven MLST loci for each isolate. The concatenated sequences were aligned using the CLUSTALW algorithm. Subsequently, the model selection was completed using the Maximum Likelihood method, and the model with the lowest Bayesian information criterion (BIC) value was selected [40]. A dendrogram was then generated using the chosen model with 1000 bootstrap replications.

### 2.8. Statistical Analysis

All of the statistical analyses were conducted using the R Statistical Environment (R Core Team). For the analysis of the predictors of hollow colonisation, only environmental data from transects with known koala home ranges (Transects 1–15, 18), opportunistic transects (Transects 16 and 17) and opportunistic sampling were used. For the analysis of the predictors of koala colonisation, only environmental data collected from transects with known koala home ranges were used (Transects 1–15, and 18).

#### 2.8.1. Predictors of Hollow Colonisation with *C. gattii*

The sun index, along with the obtained measurements of the tree height, tree diameter, hollow height from the ground level, hollow size, and tree species were used as fixed effects to generate a binomial generalized linear mixed-effects model (GLMM) with logit link function using the ‘lme4′ package to determine predictors of *C*. *gattii* colonisation of tree hollows. Hollows which were only positive for *C. neoformans* were considered negative for *C. gattii* in the analysis. We included the random effects of sites and individual trees (nested within sites). The transect was not included in the model as a random effect because many transects contained only a single sampled tree, while most contained three or fewer trees. Excluding the transect also allowed us to include opportunistic samples that were not on any transect in the model. Stepwise regression using backwards elimination was used to sequentially remove fixed effects from the full model. Small sample corrected Akaike Information Criterion (AICc) values were generated for each model produced, and the models were ranked from the lowest to the highest AICc value. The difference in AICc value (∆AICc) between each model and the model with the lowest/best AICc was also calculated. Models with a ∆AICc lower than 2 were considered equivalent to the model with the lowest AICc [41]. In the case that the second-best model had a ∆AICc lower than 2, the simpler model was chosen following the principle of parsimony. In addition, Receiver Operating Characteristic (ROC) graphs were generated and the Area Under the Curve (AUC) was calculated using the ‘pROC’ package after model selection to clarify the fit of the best model to the data.

#### 2.8.2. Predictors of Koala Nasal Colonisation with *C. gattii*

In order to account for varying transect lengths, the measurements of the number of trees positive for *C. gattii* on each transect (Variable A) and the number of hollows which were positive for *C*. *gattii* on each transect (Variable B) were standardised to a length of 100 m (or per 100 m) prior to analysis. A ‘load index’ (Variable C) was also developed to represent the ‘environmental load’ of *C. gattii* on the transect, which was derived from the total score of colony counts for the hollows sampled on the transect (score 1 = < 20 colony count, 2 = 20–100 colony count, 3 = > 100 colony count per plate) and then subsequently standardised to a transect length of 100 m.

A binomial generalized linear model (GLM) with a logit link function was produced for each of the standardised variables above in relation to whether a koala on the transect was colonised by *C. gattii*. In the case that a transect overlapped the home range of more than one koala, the information from the transect was used for each koala (Table 1). Variable B and Variable C were highly correlated (R = 0.97); hence, a GLM containing both was not tested. However, additional GLMs were produced containing Variable A and Variable B, and another containing Variable A and Variable C. The AICc values were used to determine the best model, and the ROC and AUC were generated to confirm the fit of the model.

## 3. Results

### 3.1. Mycological Culture

Over the three field trips from February to May 2017, a total of 127 environmental samples from 69 *Eucalyptus* trees were obtained from opportunistic sampling, transects within known koala home ranges (Transects 1–15, 18) and opportunistic transects (Transects 16 and 17) (Supplementary Table S1). No samples were obtained from one transect (Transect 4) due to a lack of tree hollows along this transect. Environmental samples were collected from tree hollows of six species of *Eucalyptus*: *E. albens*, *E. camaldulensis*, *E. conica*, *E. melliodora*, *E. microcarpa*, and *E. populnea*. Yeasts of the *C. gattii* and *C. neoformans* species complexes could be isolated from each of these eucalypts, with 33/69 (48%) of all of the trees sampling positive. Forty (31%) environmental samples from tree hollows were positive for *C. gattii* and/or *C. neoformans*. Sixteen (40%) of these positive tree hollow samples were isolated from transects of koalas without colonisation, while 13/40 (33%) were from transects of colonised koalas. The remaining 11/40 (27%) positive tree hollow samples were from opportunistic sampling (Supplementary Table S1). A total of 260 individual isolates were obtained from the positive tree hollow samples, ranging from one isolate to a maximum of 12 for some samples, depending on the number of colonies available for subculture from the primary plate. The environmental cryptococcal load, as a reflection of colony counts showing the brown-colour-effect on BSA plates, was similar between samples from transects with colonised koalas versus those without colonised koalas.

A total of 169 koalas were captured and sampled from the Watermark site and Dimberoy site. Of these, nine of the 169 (5%) koalas were culture positive for *C. gattii* (Supplementary Table S2). Of these nine koalas, three tested serologically positive for cryptococcal capsular antigen via latex cryptococcal antigen agglutination testing (LCAT) in addition to positive nasal colonisation, suggesting subclinical or clinical infection (data from Schmertmann et al. [42]). GPS data were available for five of these colonised koalas (DECC017, DECC051, DECC137, DECC162, and DECC198) (Supplementary Table S2).

### 3.2. Molecular Typing

Of the 260 isolates obtained from the environment, only two molecular types were detected, *C. gattii* VGI (*n* = 237) and *C. neoformans* VNI (*n* = 23). Of the positive samples, 37/40 were only positive for *C. gattii* VGI, one out of 40 was only positive for *C. neoformans* VNI, and two out of 40 were positive for both molecular types: VGI and VNI. All nine koala isolates were positive with *C. gattii* VGI for all cultures (Supplementary Table S1).

### 3.3. MLST

Twenty-eight of the environmental isolates were sequence typed and five STs were identified in the environment (Table 2, Supplementary Table S1). The most common environmental ST found at both sampling sites was ST154 (*n* = 17), followed by ST51 (*n* = 6). ST257 (*n* = 2) was detected only at the Watermark site. Two new STs were also identified: ST462 (*n* = 1) was only found at the Dimberoy sampling site, whereas ST463 (*n* = 2) was isolated from two individual trees from the Watermark sampling site.

Six STs were identified from the nine koala isolates. Both ST154 (*n* = 2) and ST51 (*n* = 3)—which were found in the environment—were also identified in samples from the nasal colonisation of koalas (Supplementary Table S2). ST159 was found in a single koala captured at the Dimberoy site. Three new STs were also found in three different koalas (ST459, ST460, and ST461) (Supplementary Table S2). These new STs had not been isolated from the environment.

Allele variation was low among the STs for most loci. The most variation was observed in the *SOD1* locus, with a total of five allele types identified (Table 2). Three allele types were evident at the *IGS1* locus. The *CAP59*, *GPD1*, *LAC1* and *URA5* loci each consisted of two alleles. The *PLB1* locus had no variation, consisting of only one allele type. The variation between allele types was evidently low, with the alleles of most loci diverging by a single nucleotide polymorphism (SNP), and only seven SNPs were identified among all of the alleles in the most variable locus, *SOD1* (Table 3).

### 3.4. Phylogenetic Analysis

**Figure 1:**
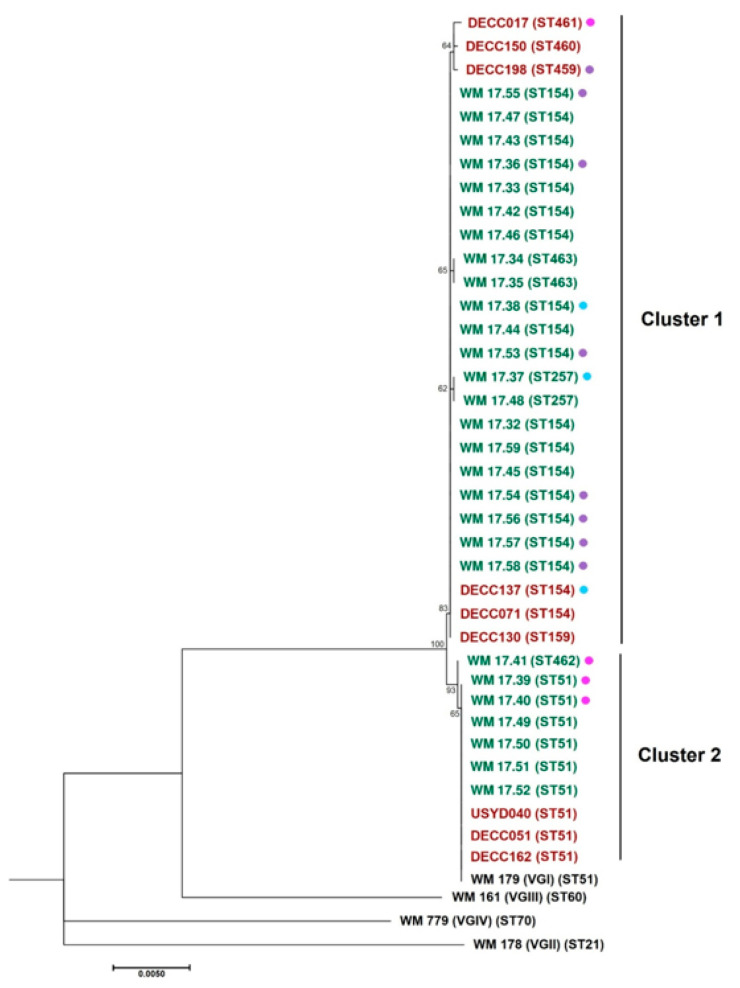
Dendrogram obtained using the concatenated sequences for all seven loci of the consensus *C. gattii*/*C. neoformans* MLST scheme for all of the isolates genotyped herein. The isolates highlighted in green are environmental, and the isolates highlighted in red are from koalas. The STs for the *C. gattii* standard reference strains are included in black. Environmental isolates obtained from a transect within the home range of a koala with a sequence typed isolate are matched using coloured circles. Bootstrap values based on 1000 replicates are shown at each node. The scale bar represents the number of substitutions per site along the branch lengths.

The dendrogram produced (Figure 1) showed two distinct clusters when analysed with the standard reference strains for the four major molecular types of *C. gattii*. Cluster 1 consists of ST154, ST159, ST257, ST459, ST460, ST461, and ST463, with ST154 appearing to be basal to the other sequence types. Notably, the isolate from one koala was grouped with the ST154 isolates, although it was identified as the ST159 sequence type. The three koala isolates with the sequence types ST459, ST460, and ST461 seem to form a cluster within Cluster 1. Overall, the analysis suggests that the other STs in this cluster may have been derived from ST154. Cluster 2 contains ST51 and ST462, which are evidently attributed to only one SNP between the two STs at the *SOD1* locus (Table 3).

### 3.5. Statistical Analysis

#### 3.5.1. Predictors of Hollow Colonisation with *C. gattii*

The step-by-step removal of the least informative fixed effects in subsequent models from the original model containing all fixed effects showed that the most predictive fixed effect was hollow size (*p* = 0.0528) (Table 4). A model containing only hollow size and random effects (Model A) was determined to be the best model when comparing AICc values. The analysis of the ROC and the AUC confirmed that the best model was a good predictor of the probability of *C. gattii* VGI in a hollow (AUC = 0.747). Probability plots for the likelihood of hollow colonisation as a function of hollow size showed that there was an approximately 20% increase in the likelihood of a hollow being colonised between a small (0–15 cm) and a large hollow (>30 cm).

GLM was used to test the effect of hollow size on the likelihood of hollow colonisation alone. In this particular model with only hollow size as a predictor for the presence of *C. gattii* in a tree hollow and no random effects, hollow size was significant (*p* = 0.04851) (Table 5), but when examining the AUC, the value was greatly reduced (AUC = 0.6023). As a result, the random effects were further investigated, and individual GLMs were produced for each random effect. Whilst the site was uninformative on the likelihood of hollow colonisation (*p* = 0.2888), the random effect of individual trees was significant (*p* = 0.0394). The significance of this random effect suggests that other variables of trees which went uninvestigated in this study exist, and that these may better predict the colonisation of hollows by *C. gattii* VGI, in addition to the variable of the hollow size.

#### 3.5.2. Predictors of Koala Colonisation with C. gattii

None of the three predictors of likelihood of koala nasal colonisation with *C. gattii* were found to be significant in GLMs containing only the individual predictors, i.e., the number of trees which were positive for *C. gattii* VGI on each transect (Variable A) (*p* = 0.3976), the number of hollows which were positive for *C*. *gattii* VGI on each transect (Variable B) (*p* = 0.2294), and the Load Sum of each transect (Variable C) (*p* = 0.1616). Similarly, none of the models containing two variables were found to be informative, i.e., Variable A and Variable B (*p* ≥ 0.3765), and Variable A and Variable C (*p* ≥ 0.2546).

## 4. Discussion

The current study provided a unique opportunity to examine the environmental presence of *C. gattii* within known home ranges of wild koalas with identified colonisation/subclinical disease status. It allowed an evaluation of the molecular links between environmental and koala isolates of *C. gattii*, and the assessment of the role of possible environmental predictors for tree hollow and koala colonisation. From the 260 environmental isolates from 69 *Eucalyptus* trees, we established *C. gattii* VGI (*n* = 237, 91%) to be the dominant molecular type in the Liverpool Plains region. *C. neoformans* VNI (*n* = 23, 9%) was also isolated from *Eucalyptus* tree hollows, but infrequently (Supplementary Table S1). All of the koala isolates were *C. gattii* VGI (Supplementary Table S2). No other molecular types were isolated from the environment or koalas. We also recorded two instances of *C. gattii* VGI and *C. neoformans* VNI sharing the same hollow. The first recorded examples of *C. gattii* and *C. neoformans* sharing the same environmental biotope were documented in a pink shower tree in Northeastern Brazil [43], but since then it has been observed from hollows in several studies [44,45], suggesting that both may be able to survive under the same conditions, despite the occurrence of a rival species suited to this niche. The significance of *C. neoformans* in cryptococcal disease in koalas is largely unknown and clinical cases are extremely rare, with the only recorded examples being a case of nasal colonisation detected in one koala from our previous study of this region [42], and a clinical case in a captive koala from Spain [46].

Relative to other countries [47,48], the environmental isolation of the *C. gattii* VGI in the Liverpool Plains—and likely the rest of eastern Australia—can be common, as shown in this study, with 31% of 127 *Eucalyptus* tree hollow samples being positive for the organism. This percentage is lower than that found previously by Schmertmann et al. 2019 (64.3%) [42] in the same region. Our experimental approach and larger sample size likely delivered a closer representation of the true prevalence of *C. gattii* VGI within the region.

A total of nine different STs, including five new STs, were observed from the two study sites. The most common ST found in the Liverpool Plains region was ST154 (61% of the sequenced isolates), followed by ST51 (21%), although worldwide ST51 is one of the most commonly isolated STs [49,50]. Whilst nine STs were isolated in total, very few differences exist between these STs, with the most variable region, *SOD1*, only containing a maximum of seven SNP differences between two alleles (Table 3). The variability of the *SOD1* locus is also expected, with multiple studies reporting the highest allele diversity in the *SOD1* locus amongst *C. gattii* isolates [51,52].

Phylogenetic analysis confirmed the close association between the STs isolated, showing strong bootstrap support for the formation of two clusters—one containing ST154 and six other STs, and another containing ST51 and ST462 (Figure 1)—suggesting clonal populations. In this case, ST154 may be the basal ST that the six other STs may have been derived from through spontaneous mutations, with the greatest difference being only three SNPs between ST154 and ST461. The isolate from the koala DECC130 was grouped within the ST154 clade, although it was identified as the ST159 sequence type. This is most likely due to the only difference between the two sequence types being a single guanine insertion at position 285 in the *GPD1* locus. In Cluster 2, ST462 may have originated from ST51, as there is only one SNP difference in the *SOD1* locus, with all of the other alleles being the same.

Of the nine koalas which were positive for *C. gattii* VGI, three were identified as ST51 and two were ST154, with both STs being the most commonly isolated from the environment for both the Watermark and Dimberoy sites. The remainder of the koala isolates were identified as novel STs, but all were closely related to either ST154 or ST51. Of these koalas, three had genotyped environmental isolates from within their home range.

**Figure 2:**
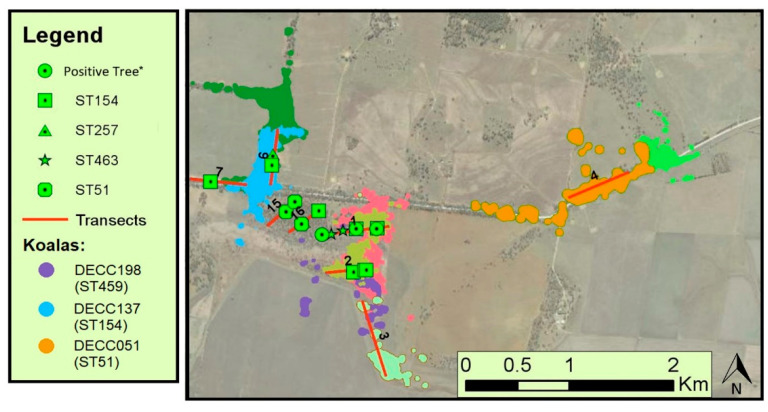
Map showing transects from the Watermark site overlaying koala home ranges with trees positive for *C. gattii* labelled, and associated sequence types provided when available. The regions highlighted in colours represent the home ranges of individual koalas. Only the koalas with nasal colonisation and sequence-typed isolates are denoted in the legend. * Positive trees from which isolates were collected but no sequence types were obtained due to sequencing issues.

**Figure 3:**
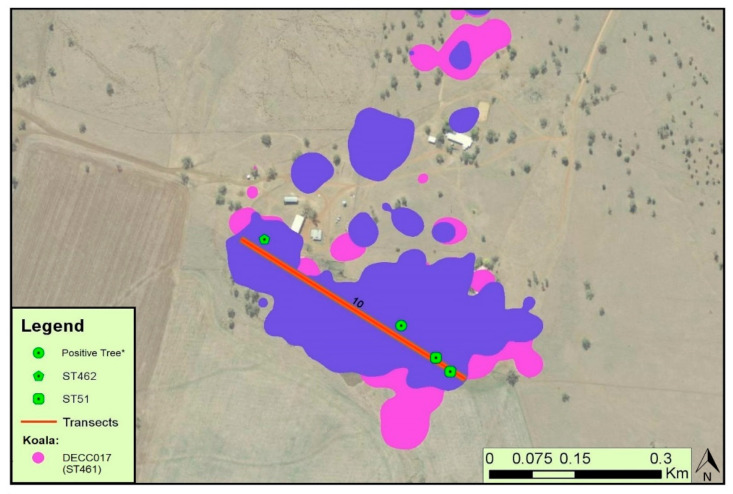
Map showing Transect 10 from the Dimberoy site overlaying koala home ranges with trees which were positive for *C. gattii* labelled, and associated sequence types provided when available. The regions highlighted in colours represent the home ranges of individual koalas. Only the koala with nasal colonisation and sequence-typed isolates is denoted in the legend. * Positive trees from which isolates were collected but no sequence types were not obtained due to sequencing issues.

A direct link between koala DECC137 on the Watermark site and the environment was observed, as ST154 was isolated from both the koala and a tree hollow within its home range (Figure 2). Similarly, koala DECC198—from which ST459 was isolated—had three trees within its home range containing the ST154 genotype (Figure 2). Although they are not the same sequence type, ST459 and ST154 are closely related phylogenetically (Figure 1), separated only by a single SNP in the *CAP59* locus, and the *SOD1* locus (Table 2). Koala DECC017—from the Dimberoy site—was found to be colonised with ST461, a sequence type within the ST154 cluster (Cluster 1), although only ST51 and the closely related ST462 were isolated from the transect within its home range (Figure 3). Considering that ST154 is the most common ST within our region, and that it was found on other transects within the Dimberoy site, it is probable that it was still present within the home range of koala DECC017. Likewise, no positive environmental isolates were obtained from Transect 4 or Transect 9, which overlay parts of the home range of koalas DECC051 (Figure 2) and DECC162 respectively, yet ST51 was isolated from both koalas. We suggest that it is highly likely that ST51 is present elsewhere within their home ranges, outside of our environmental sampling area.

From these observations, it seems that this population of free-roaming koalas is colonised by the most environmentally abundant STs and closely related genotypes of *C. gattii* VGI. The strong association between genotypes isolated from koalas and those from *Eucalyptus* tree hollows within their home range suggests that tree hollows are the likely reservoirs of *C. gattii* related to colonisation and infection. The role of *Eucalyptus* tree hollows as reservoirs for colonising isolates of koalas has important implications for the management of captive koala populations. It also indicates a risk for humans who may be frequently exposed to materials from *Eucalyptus* species, or those who work in close proximity to these trees, such as farmers, forestry workers and members of the Aboriginal community. A retrospective study by Chen et al. on clinical *C. gattii*-related cryptococcosis in Australia provides further evidence for this, with 43% of patients recalling exposure to possible environmental sources of *C. gattii.* Of these, 37% were regularly in close proximity to or working with *Eucalyptus* tree material [15].

Although some of the novel STs found only in koalas grouped together (ST459, ST460, and ST461) in our phylogenetic analysis (Figure 1), they are very closely related to ST154, and there is not enough evidence to conclude that they are strictly animal-associated strains. It is likely that these STs exist in the environment similarly to the other novel STs we identified exclusively in the environment, or they may have mutated from a basal ST and become the dominant genotype during the colonisation of the animal. ST51 and ST154 have previously been isolated globally from a range of clinical and veterinary settings (more so ST51) [49,50,53,54,55,56,57,58,59,60], but whether this infers that these two STs—and possibly their closely related novel STs found in this study—are more virulent is outside the scope of this study.

The role of *Eucalyptus* trees as the primary habitat for koalas, and the close association of both to *C. gattii* [12,17,18,19,20,21,22,61] strongly suggest that a link exists between the two. Furthermore, the linkage between the environmental and koala isolates observed in our MLST and phylogenetic analyses provides additional support for this association. Nevertheless, the statistical analysis of environmental predictors of koala colonisation found that none of the three variables representing the presence and environmental load of *C. gattii* VGI in *Eucalyptus* tree hollows were significant. It is important to note that we only had data on a small number of colonised koalas (5/19) compared to naïve koalas, and of these, no samples were obtained from the transect of one due to a lack of hollows (Transect 4), reducing the power of this statistical analysis. We are therefore unable to conclude whether there is an association between nasal colonisation in koalas and the environmental load of *C. gattii* VGI in *Eucalyptus* tree hollows across their home range.

The lack of association between the environmental load and the colonisation of koalas may not be so surprising if we consider that some koalas remain uncolonized when *C. gattii* VGI is present within their home range, and in some cases these overlap with the home ranges of colonised animals (Figure 2 and Figure 3). This may pertain to the hypothesis that the colonisation of wild koalas by *C. gattii* may not be reliant on environmental load, as observed in captive populations [22], but perhaps more so on host factors, such as behaviour and host immunity. Crowther et al. [35] infers that not all trees within a koala’s home range are utilised equally. This selective behaviour could explain the seemingly random occurrence of colonisation in our current and previous studies [42]. If we assume that nasal colonisation requires a high environmental load, as originally hypothesised, then perhaps colonisation is more likely to occur in koalas that have preferred trees within their home range that are also heavily colonised by *C. gattii*. Therefore, an approach that compares the environmental load of *C. gattii* from high-use/preferred trees of colonised koalas and naïve koalas may provide an alternative approach to the current study.

Conversely, the lack of a statistical association between the environmental load of *C. gattii* VGI and the likelihood of colonisation in koalas, as well as the absence of an animal-associated subgroup and little genetic variation—as observed from our MLST analysis of animal and environmental isolates—could infer that colonisation is reliant on host factors or even co-morbidity in koalas. This could also explain the existence of both colonised and naïve koalas in mostly overlapping home-ranges. Infection with koala retrovirus (KoRV) has been shown to be linked to the increased susceptibility of chlamydial infection [62], with KoRV being found at 100% prevalence in some populations in Queensland and New South Wales [63]. An association between KoRV infections and *Cryptococcus* species colonisation/infection is only postulated, but cannot be excluded at this point in time [62,64]. A recent investigation on the effect of KoRV on cytokine expression shows a reduced presence of IL17A, suggesting an increased susceptibility of hosts to Gram-negative bacterial and fungal infections [62]. Alternatively, the involvement of hidden host factors, similar to the presence of granulocyte-macrophage colony-stimulating factor (GM-CSF) autoantibodies in humans [7], could be an explanation for *C. gattii* colonisation/disease in seemingly healthy or KoRV-free koalas. Although we cannot be certain due to the low power of our statistical analysis, the observed abundance of *C. gattii* VGI in the environment studied, in combined with the results of our previous study, in which only 12/181 (6%) of the same koala population were colonised [42], seem to further support the contention that colonisation/infection relies more on host factors or co-infection. Future investigations into the role of the host in the susceptibility to colonisation by *C. gattii* is therefore warranted.

Our evidence suggests that the environmental load of *C. gattii* VGI in the home ranges of wild koalas has an attenuated effect compared to captive koala populations. However, the presence of *C. gattii* in the environment is still an obvious prerequisite for the colonisation of koalas. Therefore, understanding the variables that affect the presence of *C. gattii* VGI is still essential in order to provide insights into the host-pathogen-environment interaction for this organism. Our statistical analysis of predictors of hollow colonisation by *C. gattii* determined the best fitting model to contain only hollow size as a fixed effect. All of the other features of the hollow and tree from which isolates were obtained, such as the tree species, height and diameter, hollow height from ground level, and hollow directional aspect (in the form of a sunlight exposure index) were found to be uninformative. Although the *p*-value of hollow size in the best GLMM model is only 0.0528, subsequent GLM modelling and AUC analysis reinforce the significance of the hollow size on the likelihood of hollow colonisation by *C. gattii* VGI, with an approximately 20% increase in the likelihood of hollow colonisation between small (0–15 cm diameter) and large (>30 cm diameter) hollows. The random effect of trees was also found to be informative in the best GLMM model, and significant in a GLM model predicting the presence of *C. gattii* VGI in a tree hollow (*p* = 0.0394). This suggests that other variables of trees uninvestigated in this study may also be informative in the prediction of the presence of *C. gattii* VGI in tree hollows, in conjunction with hollow size.

In this study, we documented the isolation of *C. gattii* VGI from six *Eucalyptus* species, from three of which *C. gattii* had previously not been documented to the best of our knowledge: *E. conica, E. melliodora,* and *E. microcarpa*. Ellis and Pfeiffer have previously postulated a link between *E. camaldulensis* [16] and *E. tereticornis* with *C. gattii* [33], but since their early studies in the 1990s, *C. gattii* has been isolated from over 50 tree species, with most being outside of the genus *Eucalyptus* [1]. In fact, the isolation of *C. gattii* from *Eucalyptus* species other than in Australia is rare [1]. Only five of the 354 (1.4%) tree debris samples obtained from *Eucalyptus* species were found to be positive for *C. gattii* in India [65], whereas in Europe only five of the 1393 (0.4%) *Eucalyptus* trees sampled in an extensive collaborative study were found to be positive for *C. gattii* [31]. Therefore, the effect of the tree species may not be significant, as suggested in our analysis.

Considering the wide spectrum of wild animals from which members of the *C. gattii* species complex have been isolated [66], and its capacity to be distributed via contact with contaminated surfaces [67], the dispersal of *C. gattii* by wildlife—particularly by psittacine birds, which are dependent on tree hollows and are susceptible to cryptococcosis [68]—is another possible source of variation between trees and tree hollows. The inclusion of the hollow size in our statistical model supports this speculation that larger hollows would be more prone to be used by birds and larger wildlife, including koalas.

Seasonal variation in the prevalence of *C. gattii*/*C. neoformans* species complexes was not investigated as a variable for tree hollow colonisation in this study, as it does not seem to have an effect in Australia [22,42]. In contrast, multiple studies in Europe, Colombia, India, and the outbreak areas of British Columbia and the Pacific Northwest of the USA, have found that a low minimum annual temperature and high rainfall significantly limit the presence of *C. gattii* in the environment [30,31,32,69,70,71]. The dry temperate climate of NSW may therefore provide a suitable environment for *C. gattii* survival throughout the year.

The effect of other fungi in the mycobiome of *Eucalyptus* tree hollows was examined previously, and no correlation between the presence of other fungi and either *C. gattii* or *C. neoformans* was found [72]. The role of other environmental microorganisms with known fungicidal activity against *Cryptococcus* species—such as *Staphylococcus aureus* [73] and *Pseudomonas aeruginosa* [74]—and some protozoa, such as *Acanthamoeba polyphaga* [75] is yet to be examined in tree hollows but may potentially limit the presence of *Cryptococcus* species.

All-in-all, a wide range of environmental variables may influence the presence of the *C. gattii* species in the environment and tree hollows. Further exploration of these factors is needed in order to evaluate their impact and potential to predict the environmental presence of the organism.

## 5. Conclusions

Our study of *C. gattii* within a free-ranging koala population and their environment in the Liverpool Plains identified a genetic link between nose-colonising isolates in koalas and those isolated from tree hollows of *Eucalyptus* species in their home ranges. This provides further evidence for the role of tree hollows as reservoirs of *C. gattii* that cause nasal colonisation and, potentially, clinical disease in wild koala populations. Not only does this impact the future management of captive koalas, it also implies the possibility of tree hollows as a source for human infection, particularly for those that work in close proximity to or with materials from *Eucalyptus* species. Furthermore, the role of koalas as sentinels for this pathogen is emphasised. Interestingly, we could not determine an association between the environmental load of *C. gattii* VGI in tree hollows with the likelihood of nasal colonisation. However, the isolation of *C. gattii* VGI from the home ranges of naïve koalas, and in some cases, where the home ranges of naïve and colonised koalas overlap indicate that host behaviour or immunity may play a greater role in the nasal colonisation of wild koalas compared to environmental load. This is not to say that variables that affect the presence of the *C. gattii* VGI in the environment become irrelevant, as the pathogen must still exist in the environment. Our analysis of the predictors of hollow colonisation by *C. gattii* VGI determined hollow size to be a significant variable, with an opening larger than 30 cm in diameter having up to a 20% greater likelihood of being colonised by *C. gattii* VGI. This finding may have implications for the management of captive koalas, for example, avoiding the use of natural environmental enrichment with large hollows.

## Figures and Tables

**Table 1:** Transects correlated with koalas with known home ranges overlapping the transects.

Transect No.	Site	Koala ID	Koala Colonisation/Serological Status *	Sequence Type Present in the Koala	Length of Transect (m)	Load Index	No. of Trees on Transect with Hollows	No. of Hollows on Transect	No. of Hollows with *C. gattii* VGI on Transect	Sequence Type Present on Transects
1	Watermark	DECC161	Negative		560	1.5	8	12	7	ST51, ST154, ST463
2	Watermark	DECC198	C	ST459	389	1.67	4	12	8	ST154
3	Watermark	DECC058	Negative		500	0	6	11	0	
4	Watermark	DECC051	NC	ST51	437	0	0	0	0	
5	Watermark	DECC026	Negative		394	0.2	3	5	1	
6	Watermark	DECC137	SC	ST154	372	0.75	3	4	2	ST154, ST257
7	Watermark	DECC042	Negative		313	0.33	2	3	1	ST154
8	Watermark	DECC090	Negative		323	2	1	1	1	ST154
9	Watermark	DECC027	Negative		371	1	2	2	1	
9	Watermark	DECC162	NC	ST51	371	1	2	2	1	
10	Dimberoy	DECC017	NC	ST461	403	0.875	5	8	3	ST51, ST462
10	Dimberoy	DECC079	Negative		403	0.875	5	8	3	ST51, ST462
10	Dimberoy	DECC133	Negative		403	0.875	5	8	3	ST51, ST462
11	Dimberoy	DECC191	Negative		300	0	2	2	0	
12	Dimberoy	DECC180	Negative		354	0	3	3	0	
13	Dimberoy	DECC139	Negative		371	1	5	5	2	ST154
14	Dimberoy	DECC154	Negative		385	0	1	2	0	
15	Watermark	DECC140	Negative		300	1.29	3	7	3	ST51
18	Watermark	DECC015	Negative		350	1.5	1	2	1	ST154

* Results at time of capture. NC = positive nasal colonisation; SC = sub-clinical disease (asymptomatic but positive serology and nasal colonisation); C = clinical disease (positive nasal colonisation, positive serology and symptomatic); Negative = no NC, SC or C.

**Table 2:** Sequence types (ST) isolated, with the corresponding loci allele numbers, their presence in environmental or koala isolates, the number of alleles, and the total number of SNPs found at each locus.

Sequence Types	CAP59	GPD1	IGS1	LAC1	PLB1	SOD1	URA5	Environmental	Koala
**ST51**	16	5	3	5	5	32	12	✓	✓
**ST154**	16	5	3	5	5	45	12	✓	✓
**ST159**	16	14	3	5	5	45	12		✓
**ST257**	16	5	3	5	5	45	45	✓	
**ST459**	89	5	3	5	5	65	12		✓
**ST460**	16	5	12	5	5	65	12		✓
**ST461**	16	5	99	5	5	120	12		✓
**ST462**	16	5	3	5	5	119	12	✓	
**ST463**	16	5	3	58	5	45	12	✓	
**No. of Alleles**	2	2	3	2	1	5	2		
**Most SNPs between two alleles**	1 SNP	1 SNP	2 SNP	1 SNP	0 SNP	7 SNP	1 SNP		

**Table 3:** Nucleotides for each of the five *SOD1* allele types (AT) at the SNP positions in the aligned sequences.

	SNP Position						
*SOD1* Allele Type #	#401	#403	#493	#505	#508	#686	#697
32	G	T	A	A	C	C	G
45	G	A	G	G	T	A	G
65	G	A	G	G	T	A	C
119	G	T	G	A	C	C	G
120	A	A	G	G	T	A	C

**Table 4:** Coefficients and *p*-values of the two best GLMM models shown with log likelihood, AICc, and difference in AICc (∆AICc). The model containing only hollow size as a fixed effect was selected based on the principle of parsimony. Model A represents the best model, containing only the hollow size and random effects. Model B contains the hollow size and tree diameter as fixed effects as well as random effects. Model B has a ∆AICc lower than 2, and therefore is considered equivalent to the best model. Model A, containing only the hollow size as a fixed effect, was selected as the best model based on the principle of parsimony.

Model	(Intercept)	Intercept *P*	Tree Diameter	Tree Diameter *P*	Hollow Size	Hollow Size *P*	Log Likelihood	AICc	∆AICc
A	−1.9241	0.0022		-	0.4889	0.0528	−75.1	158.5097	0
B	−1.2229	0.1503	−0.0080	0.2664	0.4875	0.0492	−74.4	159.4934	0.9837

**Table 5:** Generalized linear model with only the hollow size as a predictor for the likelihood of *C. gattii* being present in a particular tree hollow. The standard error (*SE*), Z-score (*Z*) and *p*-values (*p*) are provided.

	Coefficient	*SE*	*Z*	*P*
(Intercept)	−1.9167	0.5966	−0.04478	0.00141
Hollow size	0.487	0.2469	1.937	0.04851

## Data Availability

The data presented in this study are available on request from the corresponding author.

## Supplementary Materials

The following supporting information can be downloaded at https://www.mdpi.com/article/10.3390/ijerph19084603/s1. Supplementary Table S1: List of environmental samples collected and associated metadata. Supplementary Table S2: List of positive koalas with isolated *Cryptococcus gattii* strains and associated metadata, molecular type and sequence type information.
